# A Case of Life-Threatening Bleeding Due to a Locally Advanced Breast Carcinoma Successfully Treated with Transcatheter Arterial Embolization

**DOI:** 10.3390/curroncol30020169

**Published:** 2023-02-09

**Authors:** Giulia Atzori, Raquel Diaz, Marco Gipponi, Chiara Cornacchia, Federica Murelli, Francesca Depaoli, Marco Sparavigna, Valentina Barbero, Francesco Petrocelli, Francesca Pitto, Simonetta Franchelli, Daniele Friedman, Piero Fregatti

**Affiliations:** 1 Breast Surgery Clinic, San Martino Policlinic Hospital, 16132 Genoa, Italy; 2 Department of Surgical Sciences and Integrated Diagnostic (DISC), School of Medicine, University of Genoa, 16132 Genoa, Italy; 3 IRCCS San Martino Policlinic Hospital, 16132 Genoa, Italy; 4 Department of Interventional Radiology, San Martino Policlinic Hospital, 16132 Genoa, Italy; 5 Pathology Unit, San Martino Policlinic Hospital, 16132 Genoa, Italy

**Keywords:** advanced breast cancer, embolization, bleeding

## Abstract

Locally advanced breast cancer (LABC) may rarely present with acute severe bleeding. A case report dealing with transcatheter arterial embolization to control acute bleeding in a patient with a voluminous ulcerated breast mass is described. Our findings confirm that the endovascular approach is effective in such patients in order to stabilize the patient whenever conventional treatments have failed or bleeding may be life-threatening.

## 1. Introduction

Locally advanced breast cancer (LABC) is defined as advanced non-metastatic breast cancer and includes a wide variety of clinical scenarios. LABC represents a difficult clinical problem, as most patients with locally advanced disease go on to experience disease recurrence and eventually death. Acute breast bleeding is seldom reported, although it may occur, mostly in LABC. Breast hemorrhage can be a life-threatening condition for patients, and/or it may hamper therapeutic planning. Treatment of acute bleeding of breast cancer includes compressive dressing, the use of topical or intravascular hemostatic agents, and radiotherapy. In severe cases, surgical therapy or intravascular measures may be indicated. Transcatheter arterial embolization (TAE) is a well-established option for the management of acute bleeding in patients with locally advanced or metastatic neoplasms, such as renal or liver cancers [1]. However, due to the rarity of this scenario, this therapeutic option is not usually performed in patients with acute breast bleeding. Here, the case of a patient with acute bleeding due to LABC successfully treated with TAE is reported. CARE guidelines were followed for the editing of this manuscript.

## 2. Case Report

### 2.1. Clinical Findings and Diagnostic Assessment

A 49-year-old woman was admitted to the Emergency Department (ED) with a large right breast mass; she had noticed the presence of the mass for two weeks, but she was too worried to undergo a clinical examination. A two-year earlier bilateral mammography was negative for neoplasia. Physical examination revealed a voluminous ulcerated mass with colliquated appearance occupying the entire right breast, associated with numerous small skin lesions surrounding the main mass and no signs of active bleeding; bilateral axillary lymphadenomegaly (LAM) were detected. The patient had no fever with no associated diseases. Personal and family history was negative for breast and ovarian cancer pathology. Blood tests showed increased inflammation 2 parameters (White Blood Cells = 14 × 10 9/L; CRP = 107 mg/L) and anemia (hemoglobin = 9.9 g/L). A total-body CT scan showed an “… expansive lesion with a minimal necrotic component of the right breast with a maximum diameter of 14 cm and associated skin and pectoralis muscle infiltration; ipsilateral axillary LAM with a maximum extension of 4 cm; in the right ovary, a cystic lesion of 7 cm in diameter with a solid component of heteroplastic appearance”. Distant metastases were not detected. The patient was admitted for diagnostic investigations; a punch biopsy of the primary lesion was performed with a histologic diagnosis of “Triple Negative” Infiltrating Ductal Carcinoma of the right breast with skin invasion. After a multidisciplinary discussion, the patient was deemed eligible for neoadjuvant chemotherapy (NAC) after the excision of the right ovarian lesion. This was performed through bilateral video-laparoscopic adnexectomy to exclude ovarian cancer; definitive histology diagnosed a cystic teratoma. On the second postoperative day, the patient had an episode of profuse bleeding from the breast cancer with hypotensive shock (blood pressure: 50/30 mmHg; heart rate:125 bpm) with acute anemia (Hb = 6.2 g/L). Preliminarily, sterile gauze pads soaked with tranexamic acid were applied along with chest compressing dressing with an elastic bandage to achieve hemostasis; moreover, intravenous fluid resuscitation and 50 mg of Ephedrine were given in order to support hemodynamics, and tranexamic acid 1 g, antithrombin III complex 2000 UI, and calcium gluconate 1 g were supplied plus two units of red cell suspension and one unit of fresh frozen plasma. As a result of these therapeutic measures, clinical signs improved, with a blood pressure of 100/60 mmHg and heart rate of 100 bpm. The patient was closely monitored over the next few days. Several recurrent episodes of bleeding did occur, requiring treatment with compression dressings and three other blood unit transfusions. However, the bleeding source was not effectively controlled, with Hb close to 5 g/L.

### 2.2. Therapeutic Intervention

**Figure 1:**
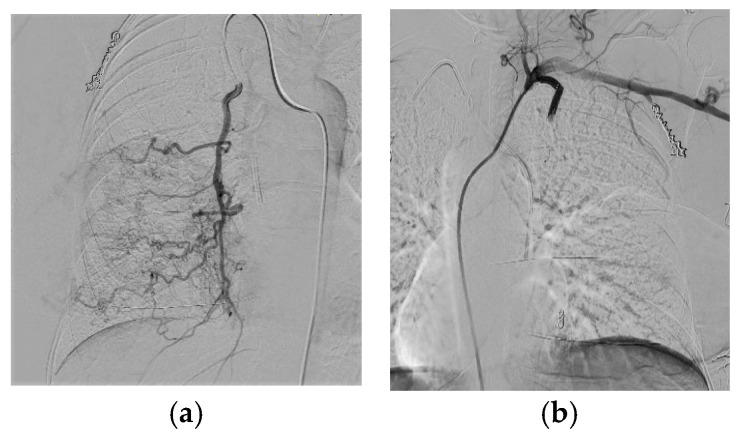
(**a**,**b**). Internal mammary artery in phase pre- (**a**) and post- (**b**) embolization. Figure 1a shows the arterial branches of the internal mammary artery responsible for breast bleeding; Figure 1b shows the disappearance of arterial branches after successful selective embolization procedure.

**Figure 2:**
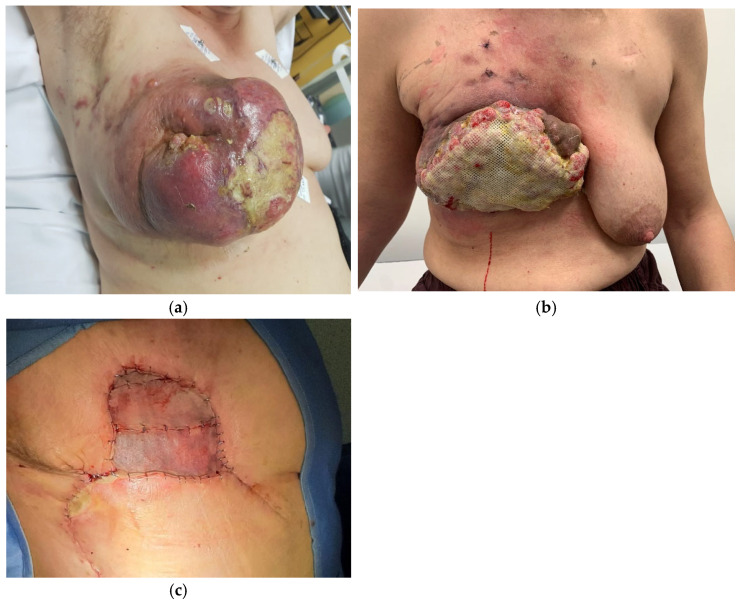
(**a**) Local conditions to admission into the Emergency Department; (**b**) Local conditions post-NAC: NAC resulted in a modest reduction in the size of the neoplasm (**c**) Local conditions post-surgery: surgery consisted of excision of the tumor, application of VAC therapy, and subsequent closure of the wall defect with a skin graft from the leg.

Due to this life-threatening clinical condition, the impossibility of starting neoadjuvant chemotherapy, and the ineffective bleeding control with conservative measures, the patient was scheduled for an interventional radiologic embolization procedure. After super-selective catheterization of the four main afferent branches to the lesion, embolization of the internal mammary artery was successfully performed by means of microspheres (Hydroperal Terumo) with a diameter of 600 microns (Figure 1). No major recurrent bleeding occurred thereafter, and Hb values gradually increased to 9 g/L. Once the clinical picture was stabilized, the patient was able to start neoadjuvant chemotherapy with Epirubicin/Cyclophosphamide 90q21 × 4, followed by weekly Carboplatin and Paclitaxel. During the period the patient was receiving NAC, there were no bleeding episodes at the breast level. The patient received medication 3 times a week to control the local situation and avoid bacterial infection. On strong suspicion of a predisposing genetic mutation for breast cancer, breast cancer gene (BRCA) 1–2 testing was performed on the patient. The genetic test demonstrated no predisposing genetic mutation. After NAC treatment, there was a partial response followed by disease progression close to the end of treatment, as confirmed by a CT scan; hence, a debulking procedure was planned in order to achieve loco-regional control. The day before surgery, the patient was re-submitted to a radiological embolization procedure to avoid profuse intraoperative bleeding caused by tumor neoangiogenesis. An imaging study of the right internal and external mammary artery showed hypertrophy of both arteries; TAE of the external mammary artery with metal coils and the internal mammary artery with metal coils and an Amplatzer vascular plug was performed. A two-stage surgical procedure was performed. First, right mastectomy with bilateral axillary lymph node dissection was accomplished, and a simple rotation flap was used to achieve partial closure of the wall defect; Vacuum Assisted Closure Therapy (VAC) was applied to the remaining wall defect for two weeks. After definitive histology confirming negative margins of excision, the staged procedure included a skin graft from the leg to cover the remaining wall defect (Figure 2).

### 2.3. Follow-Up and Outcomes

Final histological examination showed a “Triple Negative” invasive ductal carcinoma staged pT4b, N1a, G3, Mx. The patient subsequently received chest wall radiotherapy (RT). She developed partial ischemic skin flap distress that was treated with a sterile application of gauze medicated with hyaluronic acid with complete resolution of the clinical scenario. Following surgery, she developed ipsilateral upper extremity lymphedema, which was resolved with a series of physiotherapy sessions. Currently, the patient is on semiannual follow-up and has not developed local recurrences.

## 3. Discussion

Locally advanced breast cancer is a subset of breast cancer characterized by the most advanced breast tumors in the absence of distant metastasis. The definition of LABC includes tumors more than 5 cm in size with regional lymphadenopathy (N1–3), tumors of any size with direct extension to the chest wall, skin, or both (including ulcer or satellite nodules), regardless of regional lymphadenopathy, and the presence of regional lymphadenopathy (clinically fixed or matted axillary lymph nodes, or any of infraclavicular, supraclavicular, or internal mammary lymphadenopathy) regardless of tumor stage. LABC is currently estimated to account for 10% of breast cancer in women, and the subset of stage IV synchronous metastatic disease occurs in 3–4% of all diagnoses. Inflammatory breast cancer is an uncommon but highly aggressive subtype of LABC, and it is characterized by florid tumor emboli that obstruct dermal lymphatics, leading to swelling and inflammation of the affected breast. The prognosis of patients with LABC is generally poor, with a significantly high risk of local recurrence and distant metastasis, despite an aggressive surgical approach and primary radiotherapy. Treatment of LABC is multidisciplinary and involves a combination of systemic chemotherapy, surgery, and radiation therapy to optimize the possibility of a successful cure. Historically, the treatment of LABC was radical mastectomy. However, patients treated with surgery alone rapidly debulked local and systemic recurrences. LABC can be classified as an operable or inoperable disease. Patients with supraclavicular involvement, edema of the arm, satellite skin nodules, and extensive breast edema are considered inoperable. Patients treated with primary radiation therapy had a high risk of recurrence and death, as well as complications such as chest wall fibrosis, brachial plexopathy, lymphedema, skin ulceration, and skin necrosis. Neoadjuvant chemotherapy was first tested in the treatment of locally advanced breast cancer. Administration of systemic chemotherapy before definitive local therapy is advantageous for women with locally advanced disease, given that induction chemotherapy could make inoperable tumors (stage T4, N2, or N3) resectable and may increase rates of breast-conserving therapy. For women with HER-2-positive metastatic breast cancer, treatment with trastuzumab in combination with chemotherapy may improve survival. Most patients treated with induction chemotherapy show a response to therapy; 10–20% of patients achieve a complete clinical response, and 50–60% a partial response. After completing neoadjuvant chemotherapy, patients can proceed with definitive local therapy. The goal of surgery is the complete excision of the primary tumor, including loco-regional disease, and involved skin or muscles with direct extension of disease. Breast imaging, particularly Magnetic Resonance Imaging, is a useful guide for surgical planning after neoadjuvant treatments. While the traditional surgical approach was radical mastectomy, advances in systemic therapy have facilitated the downstaging of tumors, making breast-conserving surgery more common and safe even for selected patients with inflammatory carcinomas. Patients treated with surgery should receive postoperative radiation therapy to minimize the risk of local recurrence [2].

LABC rarely causes acute bleeding, resulting from the direct invasion of blood vessels by the tumor or coagulation disorders [3,4]. Some authors report sudden hemorrhage due to breast cancer localized to the mammary glands in the absence of skin invasion [5,6]. The main vascularization of the breast is the superomedial perforators of the second to fifth internal mammary arteries that arise from the internal mammary artery (IMA) and provide about 60% of the total blood supply. The thoracoacromial artery, the serratus anterior vessels, the lateral thoracic artery, and the terminal branches of the third to eighth intercostal perforators serve for the remainder of the vascular supply to the region. The IMA itself arises directly from the subclavian artery and runs along the chest wall anterior to the pleura and endo thoracic fascia, terminating at the sixth intercostal space as the superior muscular and epigastric arteries. Flow rates of up to 240 mL per minute through the IMA potentially allow the loss of 1 L of blood in a few minutes, and massive hemothorax, hemomediastinum, or pericardial tamponade may occur. The mechanism of sudden hemorrhage from a fully transected IMA has been discussed in the trauma setting, where vessel retraction in the pectoralis muscle and hemostasis can occur as a result of arterial spasm and hypotension. Resuscitation and resolution of arterial spasms can then lead to severe delayed-onset hemorrhage. Treatment of acute bleeding of breast cancer includes compressive dressing, the use of topical or intravascular hemostatic agents, and radiotherapy. In severe cases, surgical therapy or intravascular measures may be indicated [7]. Surgical ligation of the IMA by median sternotomy or lateral thoracotomy is an option to stem the massive hemorrhage originating from IMA perforators [8]. However, intraoperative difficulties may occur in locating and controlling bleeding vessels, especially in the presence of large, friable, and highly vascularized tumor masses. TAE is an interventional angiographic procedure aimed at occluding arterial or venous vessels by means of embolization agents, such as gelatin foam, polyvinyl alcohol particles, gelatin microspheres, and coils [9]. Side effects of endovascular therapy include problems of long-term patency secondary to stenosis, reduced duration of the graft, migration, infection, and endoleak. TAE is a well-established option for the management of acute bleeding in patients with locally advanced or metastatic neoplasms, such as renal or liver cancers [1]. TAE also is a procedure used to treat non-neoplastic conditions, such as iatrogenic or post-traumatic arterial bleeding. Vascular embolization is also the first-line treatment to preventatively treat pseudoaneurysms [10,11]. Endovascular therapy is rarely used to treat breast cancer-induced bleeding [12,13,14,15,16,17]. Ugras et al. [12] successfully treated bleeding due to tumor erosion of the axillary artery with the endovascular placement of a covered arterial stent. Their patient developed a life-threatening massive hemorrhage two years after cancer surgery. Manual pressure was immediately applied to the bleeding site and blood transfusion, and the patient was immediately taken to the operating room. During surgery, the bleeding was controlled with the use of a sponge stick. Angiography did not reveal any active extravasation but suggested intimal disruption, which was the origin of a probable pseudoaneurysm of the axillary artery. Given the limited surgical exposure, it was decided to place a covered stent over this defect. The placement of the stent resulted in the blockage of bleeding. The authors report that the patient had no subsequent bleeding episodes. Taib et al. [13] reported the use of TAE to treat angiosarcoma bleeding. Primary angiosarcoma of the breast is a rare condition. It is a vascular tumor, so clinically, it may have clinical onset with breast bleeding. The patient had a 6-cm hemorrhagic mass in the right breast. Angiography revealed bleeding from feeding arteries originating from branches of the left internal mammary artery and lateral thoracic artery. Embolization of the feeding vessels was performed with polyvinyl alcohol. The tumor was completely excised, and the diagnosis was a grade 2 angiosarcoma. Radiation therapy of the chest wall was performed, and investigations revealed multiple lung metastases and bone metastases. A few months after the intervention, the patient again underwent a liver embolization procedure. Additional use of TAE is transcatheter arterial chemo-embolization to achieve both embolization of the tumor and concomitant local administration of chemotherapy. Morimoto et al. [18] first described the technique of combining antitumor agents and coagulation factors for selective injection into the IMA and thoracic arteries as a pre-surgical treatment to achieve macroscopic regression of primary tumors while decreasing the toxic effects of chemotherapy.

Our patient showed a clinical picture of LABC that required immediate therapy. The presence of LABC, contralateral LAM, and an ovarian lesion deposed a strong suspicion of BRCA-related cancer, later confuted by negative genetic testing. Given the high aggressiveness of the suspected ovarian cancer, the first step was to exclude ovarian cancer pathology. The development of breast bleeding following bilateral video-laparoscopic adnexectomy surgery led to a life-threatening condition for the patient and an absolute contraindication to starting NAC. Our patient underwent TAE after conservative therapeutic measures had failed. Radiotherapy could not be used due to ulceration of the skin overlying the tumor mass. Similarly, the surgical vessel ligation with mass resection was considered a too morbid and risky procedure to be performed in this case. TAE procedure achieved stabilization of the patient clinical condition and, consequently, the possibility of starting NAC. Moreover, the TAE procedure was repeated close to surgery. TAE planning in the preoperative phase was aimed at avoiding profuse intraoperative bleeding.

## 4. Conclusions

LABC accounts for 10% of breast cancers in women, and its prognosis is generally poor. Acute breast hemorrhage is rarely reported, although it can occur. Breast hemorrhage can be a life-threatening condition for patients and can hinder therapeutic planning. The endovascular approach can be considered a safe and effective procedure in patients with acute and uncontrollable breast bleeding to stabilize their clinical condition whenever conventional treatments have failed or they are too life-threatening to be performed. TAE also can be used preoperatively to prevent profuse intraoperative bleeding in highly vascularised advanced tumors.

## Data Availability

Not applicable.

## Abbreviations

LABC	locally advanced breast cancer
TAE	transcatheter arterial embolization
ED	emergency department
LAM	lymphadenomegaly
NAC	neoadjuvant chemotherapy
BRCA	breast cancer gene
VAC	vacuum-assisted closure therapy
RT	radiotherapy
IMA	internal mammary artery
